# Robust, Breathable and Stretchable Liquid Metal Composite Nanofibre for Physiological Electrical Signal Monitoring

**DOI:** 10.1002/advs.78053

**Published:** 2026-09-27

**Authors:** Cong Huang, Zhenyu Chen, Zedong Jiang, Yuhao Jiang, Haohong Jiang, Zhenli Zhou, Yunlu Pan, Shu‐Jen Wang

**Affiliations:** ^1^ Department of Physics Hong Kong Baptist University Hong Kong China; ^2^ State Key Laboratory of Robotics and Systems School of Mechatronics Engineering Harbin Institute of Technology Harbin China; ^3^ Chongqing Research Institute of Harbin Institute of Technology Chongqing China

**Keywords:** electrospinning, health monitoring, liquid metal, nanofiber, physiological Signal, wearable electronics

## Abstract

Conductive elastic composite materials possess great application potential in the field of bioelectronic devices due to their excellent stretchability and electrical conductivity. However, conventional electronic skins hardly integrate the core characteristics of human natural skin simultaneously, including air permeability, water resistance, and high mechanical strength. In this work, a flexible and stretchable electronic skin is fabricated by combining liquid metal and electrospinning technology. Its resistance variation is less than 20% under stretching, twisting, and other mechanical deformations, while its water vapor transmission rate reaches 8100 g/m^2^·d. The electrospun fibrous structure can effectively suppress liquid metal leakage and delivers an electromagnetic interference shielding effectiveness of over ‐10 dB in the 1–18 GHz frequency range. Benefiting from these superior properties, the material enables comfortable and stable collection of various physiological electrical signals, maintaining reliable sensing performance even in humid environments and electromagnetic interference scenarios. This composite fabrication strategy remarkably improves the stability and reliability of biological monitoring in practical application scenarios.

## Graphical abstract

A conductive TPU/liquid‐metal nanofibrous film is fabricated via ultrasonic dispersion, electrospinning, and mold imprinting. The film is breathable, stretchable, deformation‐stable, and provides electromagnetic shielding. n‐Butanol encapsulation mitigates liquid‐metal surface tension and improves biocompatibility. Stable ECG, EOG, EEG, and EMG signals are obtained under sweating and electromagnetic interference, supporting high‐performance e‐skin health monitoring.

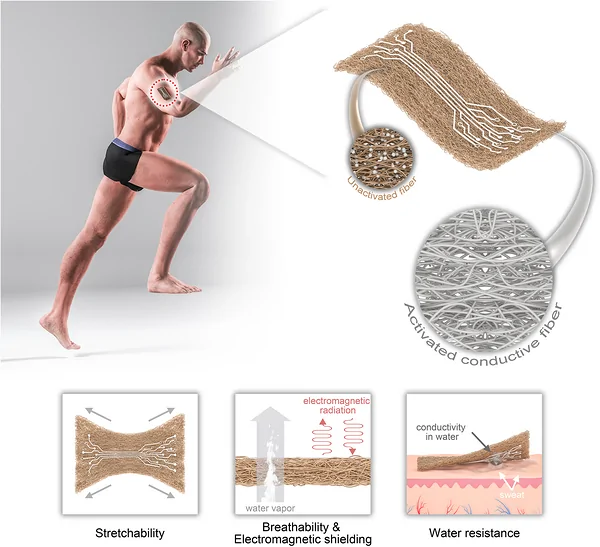

## 1 Introduction

Elastic conductive materials play a critical role in the development of next‐generation biointerfacing electronic skin, with broad applications in implantable devices [1, 2, 3, 4, 5], optoelectronic devices [6, 7, 8], neural devices [9], and soft robotics [10].

The complex environments encountered during physiological monitoring necessitate that electronic devices maintain reliable performance under dynamic deformation. Previous research has focused on two primary strategies: 1) structural designs that impart extensibility to rigid materials [11, 12], and 2) the development of intrinsically stretchable materials [13, 14, 15]. Although these approaches have significantly advanced the field of stretchable conductive elastomers, they still encounter critical limitations, including: 1) the elastic modulus mismatch between rigid‐material‐based stretchable structures and biological interfaces, and 2) the inferior electrical conductivity inherent in organic‐based intrinsically stretchable conductors.

Furthermore, during electrophysiological measurements, human skin requires continuous moisture evaporation to maintain stable and comfortable physiological conditions. Electronic devices fabricated on solid substrates can impede the skin's self‐regulatory processes, leading to complications such as skin irritation and inflammation after prolonged wear. Concurrently, the accumulation of sweat can compromise the interfacial adhesion between electronic components, thereby undermining overall device reliability. By integrating electrospinning technology [16], the porosity of electronic skins can be significantly enhanced. When coupled with electrospraying, conductive materials—such as liquid metals [17, 18] and nanoparticles [19]—can be uniformly deposited onto the surface of nanofibers to produce highly stretchable and breathable conductive fibers. However, the instability of the spraying process and the complexities of fabrication continue to hinder the advancement of flexible electronic skins.

This study presents a robust, breathable, and stretchable liquid metal‐polymer composite nanofiber electronic skin (RBS‐LMS). By employing ultrasonication and high‐voltage electrostatics to modulate the distribution of liquid metals, coupled with imprinting process, we can achieve a controllable circuit design. This approach mitigates the liquid metal leakage problem inherent in conventional liquid metal spraying processes. The electrical resistance of the RBS‐LMS exhibited a variation of less than 20% under various mechanical deformations, including bending (radius of 1 mm), twisting (180°), and stretching (80%). The electrical conductivity can reach 13440.86 S·m^−1^. Furthermore, it achieves a peak electromagnetic interference shielding effectiveness of ‐10 dB across the 1–18 GHz frequency band, effectively minimizing noise interference during electrophysiological monitoring. Notably, the synergistic combination of stretchability, breathability, and underwater conductivity—alongside a robust tensile strength of 744.92 kPa—ensures superior user comfort for physiological signal acquisition (e.g., electrocardiography (ECG), electromyography (EMG), electrooculography (EOG), and electroencephalography (EEG)) in complex, diaphoretic environments. The facile processability, mechanical resilience, high electrical conductivity, and exceptional breathability and water resistance of RBS‐LMS underscore its potential to significantly advance the field of wearable electronics.

## 2 Results

### 2.1 Structural and Operational Schematic

**FIGURE 1:**
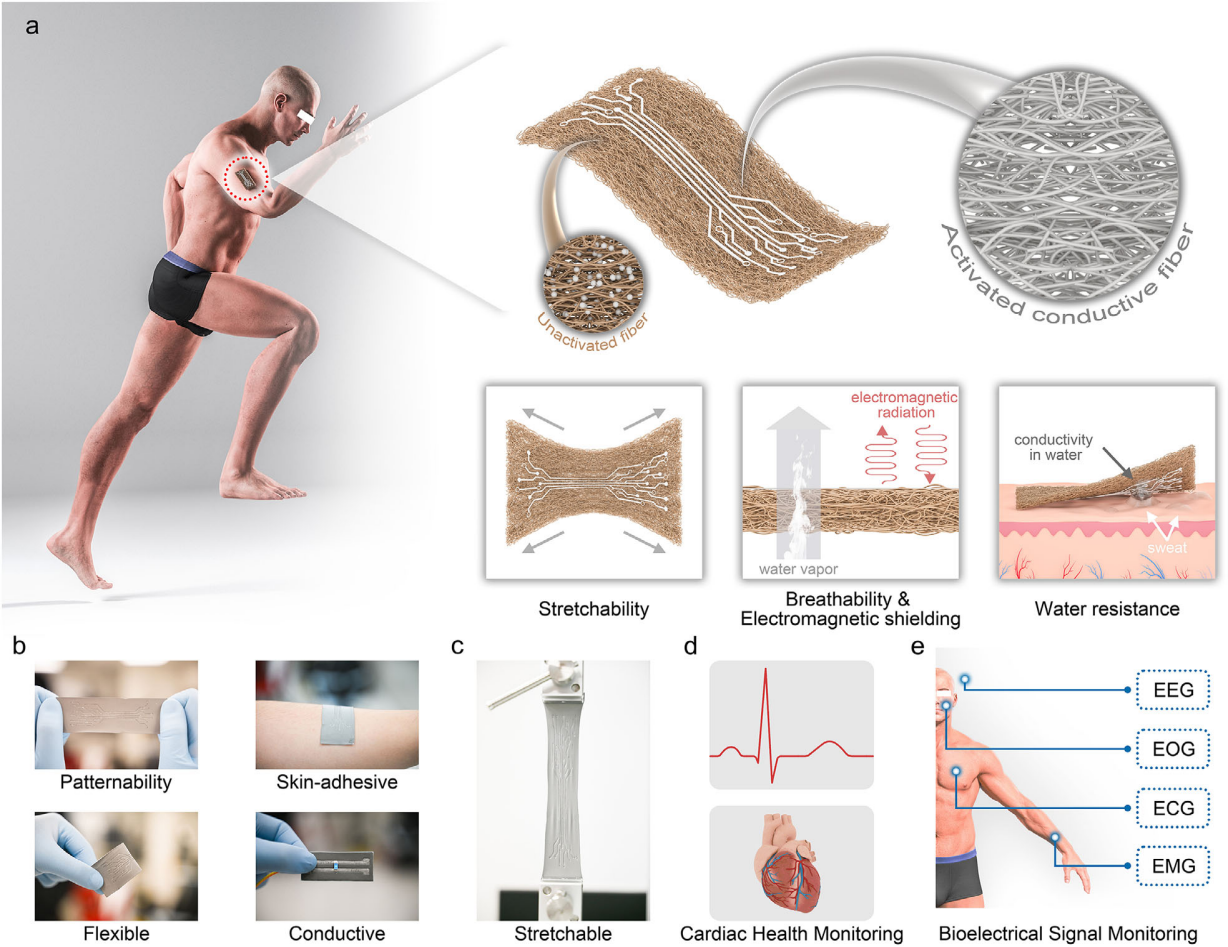
Structural design and application prospects of the RBS‐LMS based on thermoplastic polyurethane and liquid metal. (a) Composition of the RBS‐LMS, including the pristine and activated regions. (b) Optical images of the RBS‐LMS, highlighting its capabilities for patterning, physiological attachment, flexibility, and conductivity. (c) The stretching process of the RBS‐LMS. (d) Schematic illustration of electrocardiogram (ECG) monitoring. (e) Measurement of various electrophysiological signals using the RBS‐LMS. Schematic generated with 3ds Max and Photoshop.

RBS‐LMS primarily consists of a liquid metal conductive phase inside an insulating elastic TPU scaffold. (Figure 1a). The as‐deposited RBS‐LMS film are non‐conductive and may become electrically conductive through mechanical activation. Owing to the incorporation of stretchable polyurethane as the structural framework, the RBS‐LMS possesses inherent stretchability. Simultaneously, the interstices within the fibrous network provide channels for the diffusion and evaporation of gases and liquids. Utilizing 3D‐printed circuit molds allows for the facile patterning of the RBS‐LMS (Figure 1b). Compared with other fiber‐processing strategies, such as coating and spraying, directly dispersing liquid‐metal microparticles into the spinning solution for subsequent spinning enables the resulting RBS‐LMS membrane to exhibit improved resistance to liquid‐metal leakage (Figure S1). Meanwhile, the fibrous architecture inherently provides high air permeability, which is beneficial for biocompatibility during long‐term wear (Figure S2). More importantly,The flexibility, conductivity, and intrinsic stretchability of the RBS‐LMS (Figure 1c) render it a potential candidate for monitoring electrophysiological signals, such as electrocardiography (ECG), electromyography (EMG), electrooculography (EOG), and electroencephalography (EEG) (Figure 1d,e).

### 2.2 Fabrication Process and Materials Characterization

**FIGURE 2:**
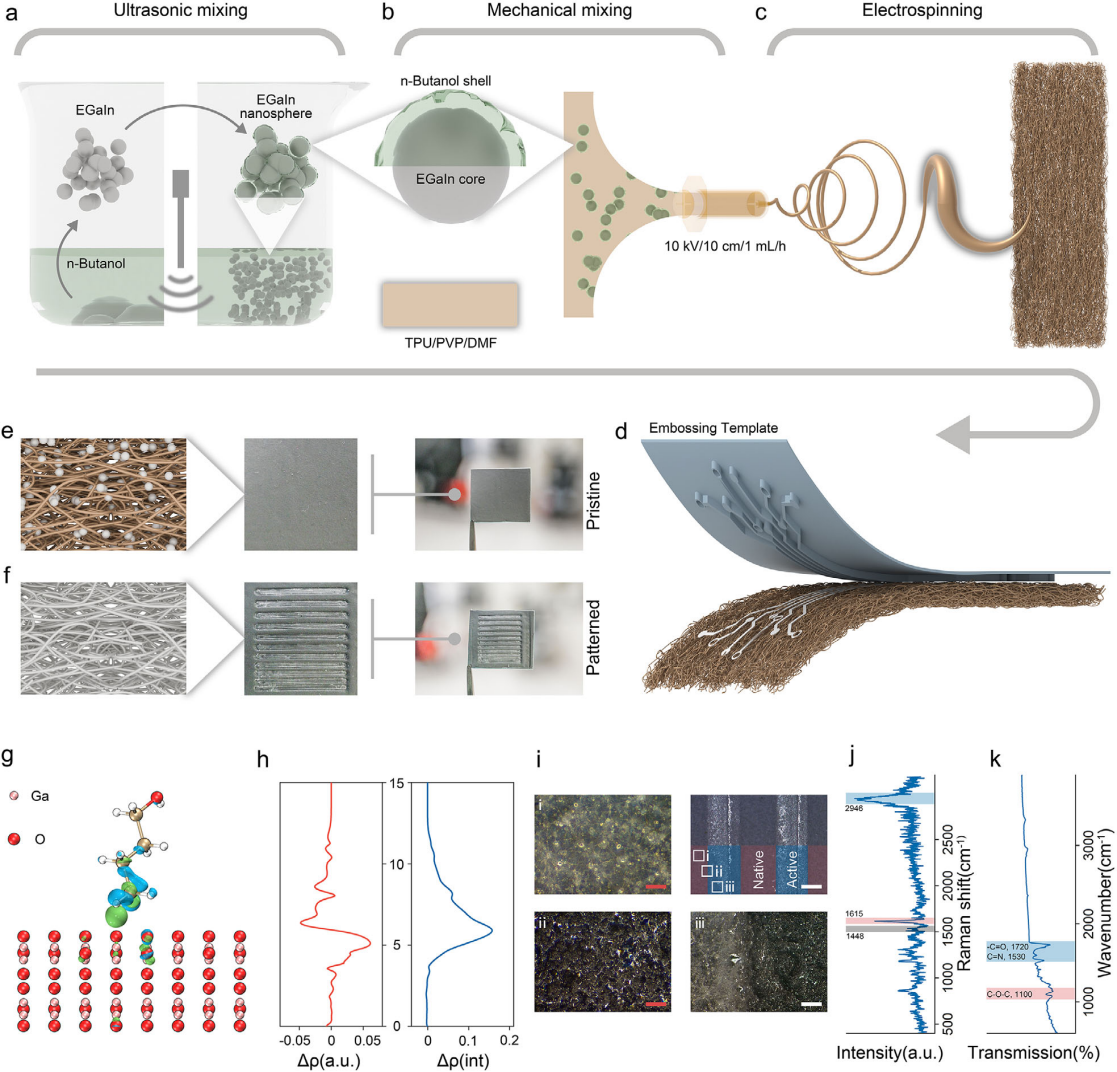
Processing procedure and characterization of RBS‐LMS. (a) Dispersion process of liquid metal. (b) Mechanical mixing of liquid metal and polyurethane. (c) Fabrication of the initial RBS‐LMS via electrospinning. (d) Activation of the initial RBS‐LMS using imprinting technology. (e) Initial state of RBS‐LMS. (f) Activated state of RBS‐LMS. (g,h) Differential charge density map between liquid metal and n‐butanol. (i) Microscopic image of RBS‐LMS. (j) Raman spectrum of RBS‐LMS. (k) FTIR spectrum of RBS‐LMS. Schematic generated with 3ds Max and Photoshop.

The fabrication of RBS‐LMS mainly consists of three parts: liquid metal dispersion, electrospinning process, and customized circuit imprinting. First, liquid metal was mixed with n‐Butanol solution, and then an ultrasonic probe was immersed into the liquid to disperse the solution at a power of 200 W (Figure 2a). Under the action of ultrasound, the liquid metal was rapidly dispersed into small particles and uniformly distributed in the n‐Butanol solution (Figure 2b). In the next step, the liquid metal dispersion was mixed with TPU/PVP/DMF solution, followed by the electrospinning process to form a uniform electrospun film (Figure 2c). The fabricated electrospun film did not possess electrical conductivity (Figure 2d), because the liquid metal was in a discontinuous dispersion state in the electrospun fibers at this stage, failing to form a conductive pathway (Figure 2e). A relief circuit template was fabricated using 3D printing technology and pressed onto the surface of the electrospun film. After applying pressure, the originally dispersed liquid metal particles were broken, thereby covering the surface of the electrospun fibers and forming a filamentous conductive network (Figure 2f).

To further determine the resolution of the imprinting processing technology, imprint templates with spacing ranging from 50 µm to 1000 µm were fabricated and subjected to imprinting processing. The results indicate that the minimum imprinting resolution can reach 100 µm, which initially meets the printing requirements for customized circuits.

During the fabrication process of RBS‐LMS, the liquid metal dispersion process affects the stability of the electrospinning process.

Compared with the strategy using ethanol as the dispersion medium [20], the liquid‐metal microparticles in RBS‐LMS exhibit a much smaller diameter of only 149 nm, compared with 801 nm when ethanol is used. The smaller liquid‐metal particle size facilitates their dispersion within the electrospun layer (Figure S3). More importantly, the smaller particle size favors the maintenance of the dispersed state of the liquid‐metal microparticles in the solution. Liquid‐metal microparticles with larger diameters are more prone to aggregate into larger particles and subsequently sediment in the solution (Figure S4).

First‐principles calculations [21] reveal a significant charge transfer phenomenon when liquid metal comes into contact with n‐Butanol (Figure 2g). A distinct charge transfer is observed between Ga atoms in the liquid metal and H atoms in n‐Butanol (Figure 2h). This charge transfer induces the formation of weak interactions between liquid metal and n‐Butanol, and an obvious weak interaction region (Figure S5) can be observed in the charge transfer area. Meanwhile, Hirshfeld charge analysis (Figure S6) and H charge analysis (Figure S7) between n‐Butanol molecules indicate that the H‐H bond interactions between adjacent n‐Butanol molecules further stabilize the liquid metal dispersion.

After the RBS‐LMS film undergoes imprinting processing, its surface morphology undergoes significant changes (Figure 2i). If a relatively high imprinting intensity is applied, a small portion of liquid metal will leak, forming obvious granular reflective regions. Furthermore, Raman spectroscopy (Figure 2j) and FTIR (Figure 2k) were employed to confirm the composition of the RBS‐LMS film.

For RBS‐LMS, the surface is predominantly composed of electrospun TPU fibers. The characteristic peaks at 2946 and 1448 cm^−^^1^ are attributed to the stretching and bending vibrations of ─CH_2_, respectively. The characteristic peak at 1615 cm^−^^1^ is attributed to the symmetric stretching vibration of C─C bonds (Figure S8). Similarly, in the FTIR spectrum, the characteristic peaks at 1148 and 1100 cm^−^^1^ correspond to hydrogen‐bonded C─O─C and free C─O─C, respectively. The characteristic peaks at 1530 and 1720 cm^−^^1^ correspond to C═N and ‐C═O, respectively (Figure S9).

### 2.3 Mechanical Properties

**FIGURE 3:**
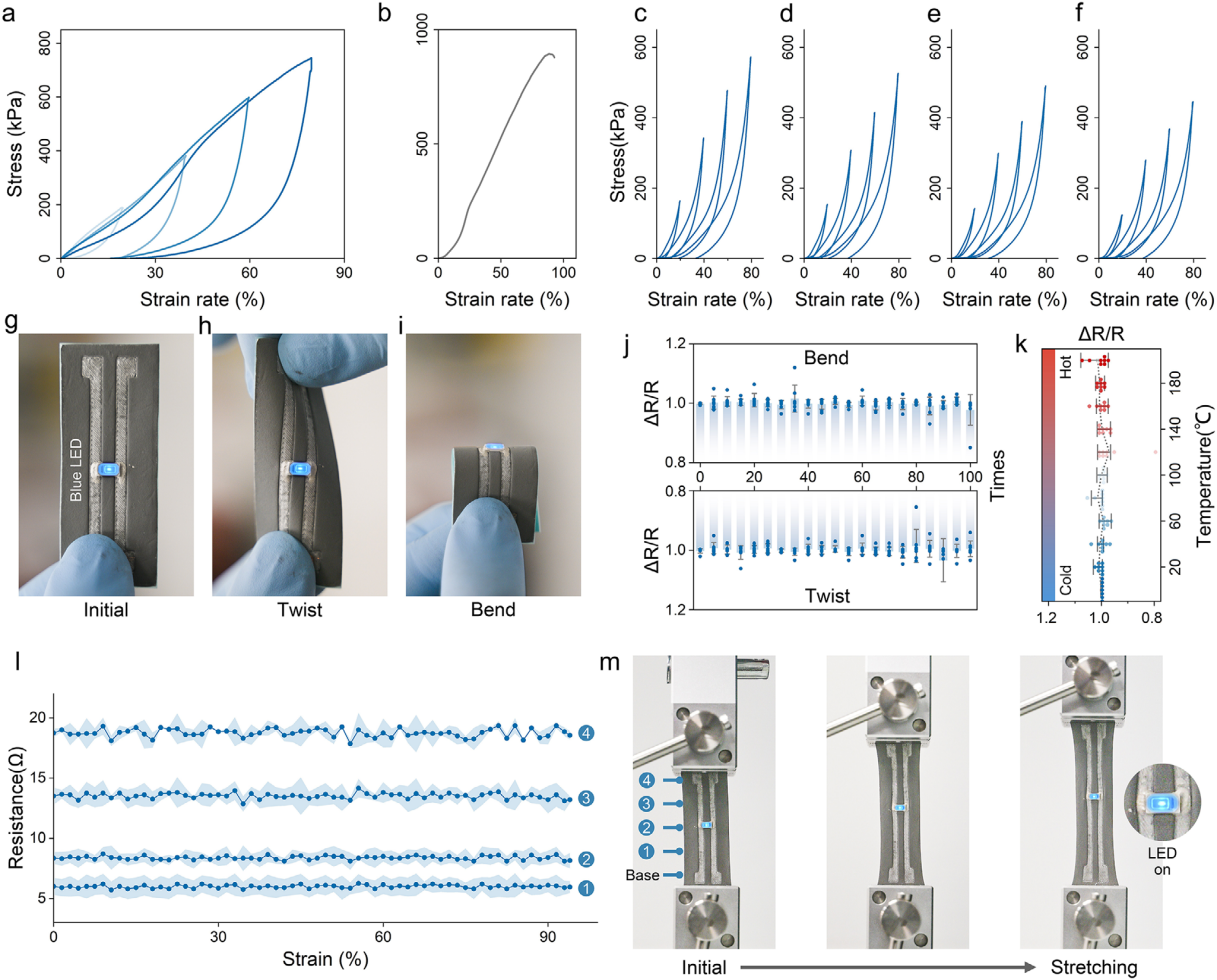
Mechanical properties of RBS‐LMS. (a) Stress‐strain curves of RBS‐LMS under 20%, 40%, 60%, and 80% strains. (b) Tensile‐fracture diagram of RBS‐LMS. (c–f) Stress loading curves of RBS‐LMS after 5, 10, 15, and 20 strain cycles, respectively. (g–i) Photographs of RBS‐LMS after patterned activation, as well as after torsion and bending. (j) Resistance changes of RBS‐LMS during bending and torsion processes. (k) Effect of temperature on RBS‐LMS. (l) Resistance change diagram of RBS‐LMS during the stretching process. (m) RBS‐LMS can still maintain a conducting state during the stretching process. Schematic generated with 3ds Max and Photoshop.

Both TPU and liquid metal, the main components of RBS‐LMS, exhibit excellent stretchability, which is beneficial for wearable applications. Bulk RBS‐LMS was fabricated into a sample with dimensions of 5 × 2.35 × 0.06 mm. A loading rate of 0.2 mm/s was adopted for the tensile test. Based on the strain rate at fracture, the calculated tensile strengths at strains of 20%, 40%, 60%, and 80% were 189.32, 382.27a, 598.07, and 744.92 kPa, respectively (Figure 3a). It can be observed that RBS‐LMS exhibits a distinct hysteresis phenomenon when the stress is released, indicating its elastic hysteresis characteristic. RBS‐LMS fractures directly when the stress reaches 894.20 kPa (Figure 3b). To further investigate the effect of tensile cycles on RBS‐LMS, cyclic tensile tests with 5, 10, 15, and 20 cycles were performed, respectively. The results show that RBS‐LMS still demonstrates good elastic recovery after 20 tensile cycles. The recovery stress strengths at 20% strain were 163.76, 153.62, 141.79 kPa, and 123.55 kPa for 5, 10, 15, and 20 cycles, respectively, while those at 80% strain were 573.18, 523.91, 490.57, and 445.65 kPa (Figure 3c–f). These results indicate that RBS‐LMS possesses certain elastic hysteresis and deformation resistance capabilities.

In physiological measurement applications, to conform to the uneven skin surface or adapt to joint movements, the device is required to maintain stable electrical properties under deformation conditions. Taking the original state as the reference (Figure 3g), two types of deformations—torsion (Figure 3h) and bending (Figure 3i)—were applied to RBS‐LMS, respectively. It can be observed that after approximately 100 deformation cycles, the average resistance change rate under bending is 0.15, and the average resistance change rate under torsion is approximately 0, demonstrating the deformation tolerance of RBS‐LMS (Figure 3j). When RBS‐LMS was heated from room temperature to 190°C, and the resistance was continuously measured during the heating process, with a resistance change rate of only 0.07 (Figure 3k). These results indicate that the resistance of RBS‐LMS can remain stable throughout the heating process.

Although RBS‐LMS can maintain electrical stability under torsion and bending deformations, extreme stretching conditions may also occur in human physiological applications. Therefore, the resistance of RBS‐LMS was continuously measured during the stretching process. Four measurement points were taken at equal intervals, and as the stretching rate increased, the resistance of RBS‐LMS remained basically stable, with the change rates of the four measurement points being 0.01, 0.03, 0.03, and 0.02 (Figure 3l and Figure S10), respectively.

Before stretching, the surface of RBS‐LMS is relatively smooth. After being stretched to 80%, deformation of part of the fibrous structure can be observed (Figure S11). After recovery from stretching, a small amount of liquid‐metal particles may remain on the surface owing to compression within the internal fibrous structure. Overall, mechanical deformation has only a limited effect on the performance of RBS‐LMS, suggesting its potential for applications in wearable electronics.

When the stretching length is 3 cm, the measured width and thickness of the conductive path are 3 and 0.04 mm (activated), respectively. The corresponding resistance is 18.6 Ω, and the calculated electrical conductivity is approximately 13440.86 S·m^−1^. It can be observed that during the stretching process, the LED lamp remained lit continuously, and no visible change in brightness was observed (Figure 3m). These results all indicate that RBS‐LMS can maintain stable electrical properties under various deformation conditions.

### 2.4 Electromagnetic Shielding Performance

**FIGURE 4:**
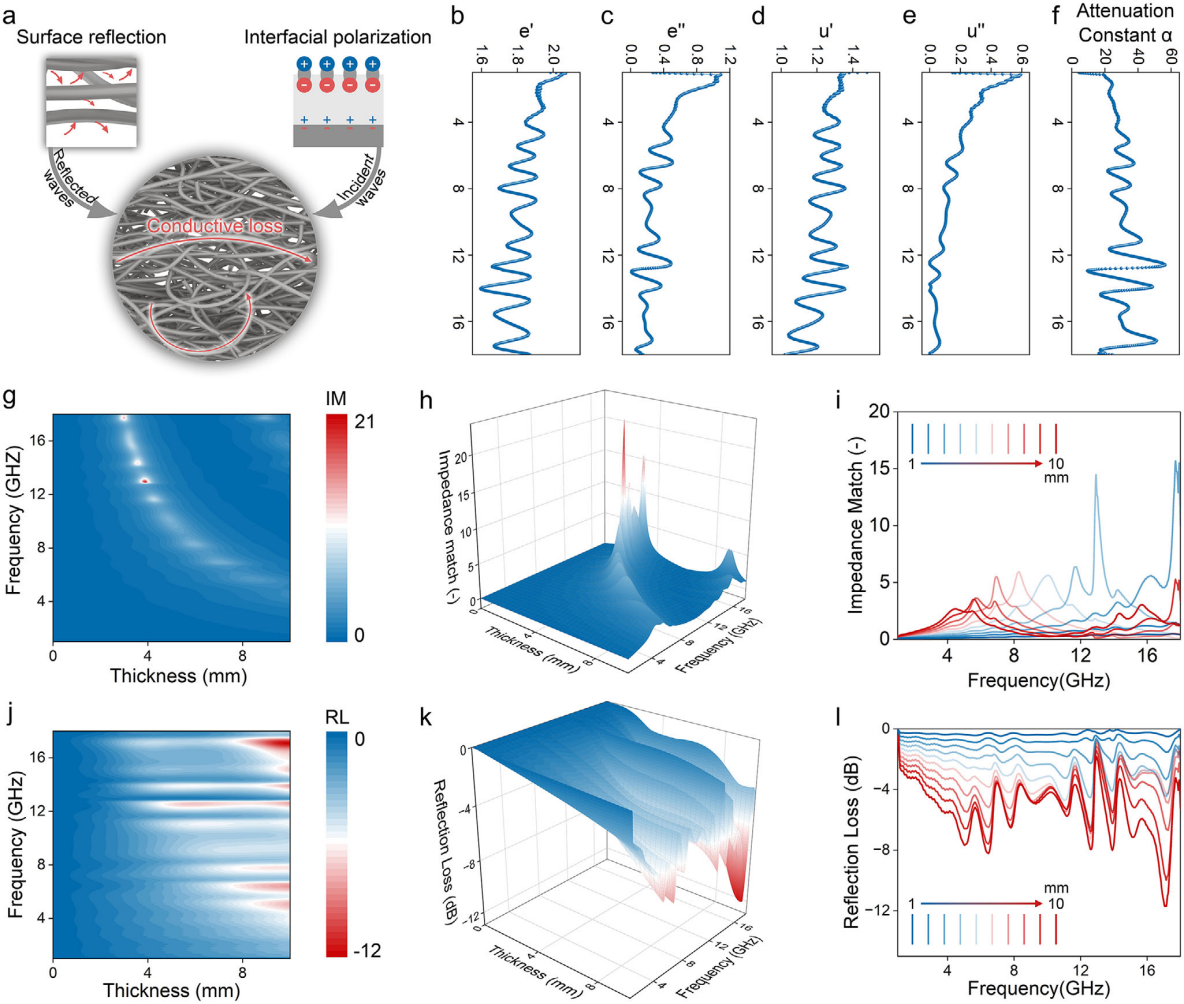
Electromagnetic shielding performance of RBS‐LMS. (a) Schematic diagram of electromagnetic wave reflection and absorption by RBS‐LMS. (b–e) Variations of permittivity and permeability. (f) Variation of the attenuation constant. (g,h) Electromagnetic signal absorption by RBS‐LMS with different thicknesses. (i) Effect of RBS‐LMS thickness on the electromagnetic signal absorption range. (j‐k) Electromagnetic signal reflection by RBS‐LMS with different thicknesses. (l) Effect of RBS‐LMS thickness on the electromagnetic signal reflection range. Schematic generated with 3ds Max and Photoshop.

During physiological electrical measurements, various low‐frequency (alternating current) and high‐frequency (WiFi, Bluetooth) signals in the environment may interfere with the sensor [22]. The liquid metal‐electrospun fiber structure of RBS‐LMS can synergistically reduce electromagnetic interference through two modes: absorption and reflection (Figure 4a). Signals with a frequency range of 1–18 GHz were selected as interference signals. Within the entire frequency range, the real part of the permittivity (ε′) was maintained at 1.7–2.0 (Figure 4b), exhibiting relatively stable polarization capability, while the imaginary part (ε″) gradually decreased with increasing frequency (Figure 4c), indicating that energy dissipation is mainly derived from the dielectric loss mechanism. Meanwhile, both the real part (µ′) (Figure 4d) and the imaginary part (µ″) (Figure 4e) of the permeability fluctuated slightly around 1, suggesting that RBS‐LMS has a weak magnetic response and makes a limited contribution to the overall attenuation process. This difference between dielectric and magnetic responses further reflects the dominant role of the dielectric mechanism in RBS‐LMS. Overall, its attenuation constant (α) remained at a high level throughout the entire frequency band and showed a significant enhancement in the 12–16 GHz range (Figure 4f). This characteristic indicates that electromagnetic waves can be effectively dissipated after entering the interior of RBS‐LMS, and also reflects a certain frequency‐dependent attenuation property.

For the electromagnetic signal absorption part, as the thickness increases, the matching region shifts gradually toward the low‐frequency direction (Figure 4g,h), showing an obvious thickness‐dependent characteristic. This change is consistent with the quarter‐wavelength matching mechanism and also reflects the variation of electromagnetic wave propagation conditions inside the RBS‐LMS. Meanwhile, the matching state varies under different thickness conditions (Figure 4i), indicating that the interaction between electromagnetic waves and RBS‐LMS can be continuously adjusted with the change of structural parameters.

For the reflection part of electromagnetic signals, there are multiple absorption peaks within the test frequency band (Figure 4j), and effective absorption (RL < −10 dB) can be achieved under different thickness conditions. As the thickness increases, the absorption peaks shift gradually toward the low‐frequency direction, showing a trend of continuous variation (Figure 4k). The minimum reflection loss of RBS‐LMS is approximately −12 dB, indicating that it can maintain a relatively stable attenuation behavior under different thickness conditions. Meanwhile, the distribution of multiple absorption peaks within a wide frequency band (Figure 4l) also reflects the adaptive characteristics of RBS‐LMS under the action of electromagnetic waves with different frequencies.

The preceding investigations were conducted using RBS‐LMS with a thickness of 60 µm in a partially activated state. To further investigate the effects of thickness and activation state on electromagnetic shielding performance, two groups of RBS‐LMS samples were prepared in the unactivated and activated states, respectively. Each group consisted of RBS‐LMS samples with three different initial thicknesses of 60, 120, and 180 µm. As the thickness increased from 60 to 180 µm, the electromagnetic shielding performance of the unactivated RBS‐LMS increased by a factor of 3.88 across the entire frequency range (Figure S12). In the activated state, the electromagnetic shielding performance increased by a factor of 1.03. These results indicate that the electromagnetic shielding performance increases with increasing thickness. Comparison between the unactivated and activated states shows that the average electromagnetic shielding effectiveness of unactivated RBS‐LMS is less than 1 dB, because the liquid‐metal particles remain disconnected and cannot form effective conductive pathways. In the fully activated state, the shielding effectiveness increases to 30–60 dB. These results indicate that a higher degree of activation produces more extensive conductive pathways within RBS‐LMS, thereby improving its electromagnetic shielding performance.

Wearable electronic devices are continuously exposed to mechanical deformation and sweat‐induced degradation during practical use. Four groups of samples were prepared, including pristine RBS‐LMS, RBS‐LMS immersed in artificial sweat for 120 h, RBS‐LMS subjected to 100 bending cycles at 60°, and RBS‐LMS subjected to 100 twisting cycles at 90° (Figure S13). Interestingly, the electromagnetic shielding performance of RBS‐LMS increased after liquid immersion and mechanical deformation, which may be associated with passive activation occurring during deformation. Overall, RBS‐LMS exhibits resistance to liquid exposure and a certain degree of mechanical deformation, suggesting its potential for future wearable electronic applications.

### 2.5 Anti‐Leakage, Air Permeability, Underwater Conductivity, and Recyclability

**FIGURE 5:**
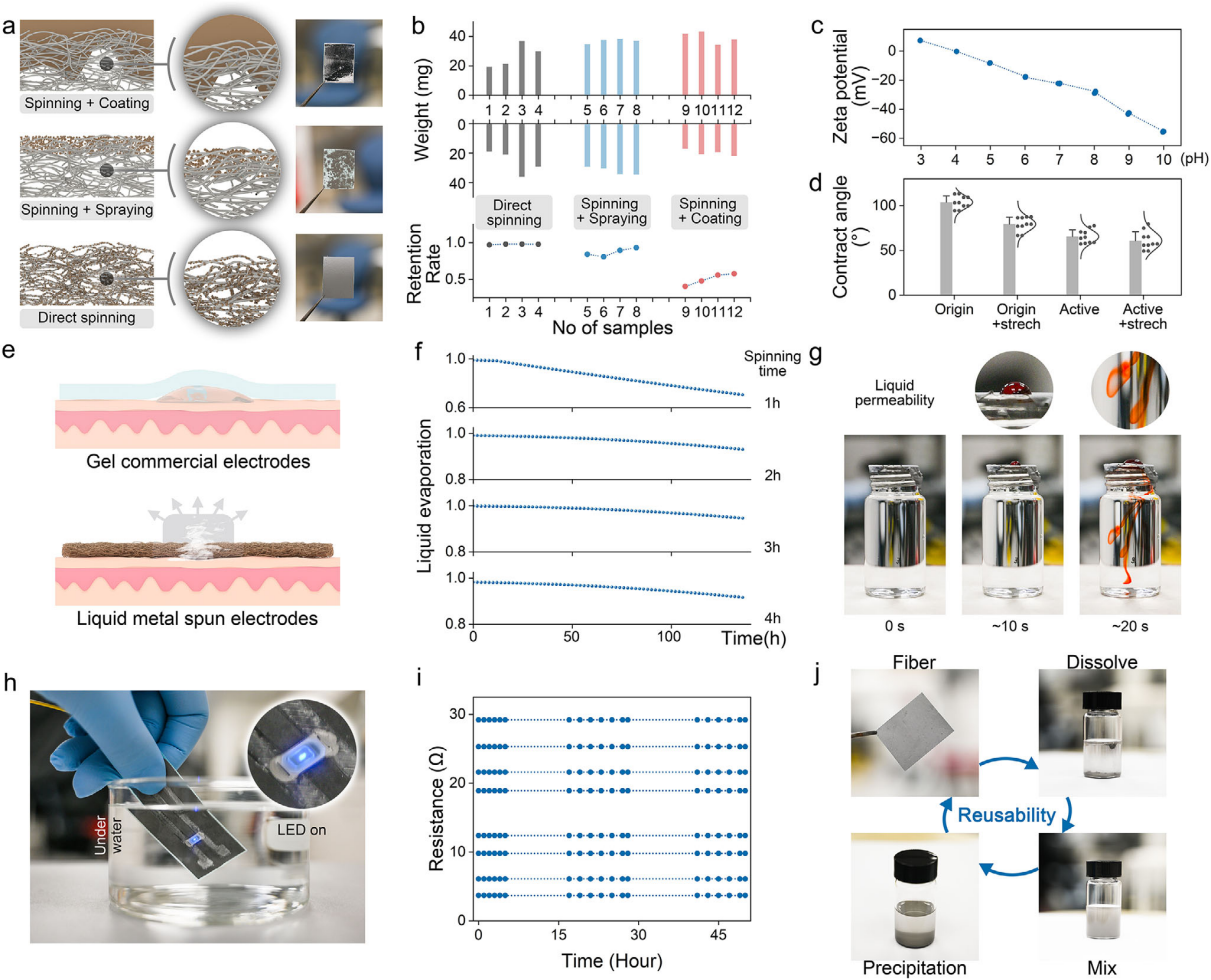
Anti‐leakage, air permeability, underwater conductivity, and recyclability of RBS‐LMS. (a) Comparison of conductive structures obtained using different processing methods. (b) Comparison of leakage amounts under different processing methods. (c) Zeta‐potential measurement of RBS‐LMS. (d) Influence of mechanical deformation and activation state on the surface wettability of RBS‐LMS, as evaluated by contact‐angle measurements. (e) The spinning structure of RBS‐LMS can allow the penetration of gases and solutions. (f) The evaporation rate of RBS‐LMS. (g) The liquid permeability of RBS‐LMS. (h) The underwater conductivity of RBS‐LMS. (i) The resistance change of RBS‐LMS after long‐term storage underwater. (j) The recycling steps of RBS‐LMS. Schematic generated with 3ds Max and Photoshop.

The development of conductive fibrous materials has involved two representative strategies: directly coating liquid metal onto the surface of electrospun fibers and spraying dispersed liquid‐metal particles onto the fibrous surface (Figure 5a). In the direct‐coating approach, liquid metal is applied without additional treatment and directly adheres to the fibrous surface to form conductive pathways. In the spraying approach, dispersion of the liquid metal improves its adhesion to the fibrous surface. However, both approaches suffer from substantial liquid‐metal leakage when subjected to external friction. As shown in Figure 5b, the mass loss of all four RBS‐LMS samples remained below 2.90% after friction was applied to the fibrous surface under a load of 5 N. In contrast, the four films prepared by coating and spraying exhibited mass losses of 49.5% and 13.1%, respectively, after being subjected to the same level of friction. These results demonstrate that the optimized RBS‐LMS film exhibits effective resistance to liquid‐metal leakage, which is important for improving the comfort of wearable devices.

To further investigate the surface characteristics of RBS‐LMS, zeta‐potential and contact‐angle measurements were performed. As the pH increased, the zeta potential became progressively more negative, indicating an increased degree of deprotonation of surface functional groups (Figure 5c). The isoelectric point was located between pH 3 and 4. The consistency of the gap height during measurement confirmed the reliability of the obtained results (Figure S14). In wearable electronic devices, the contact angle can influence the behavior of sweat on the material surface [23]. For the contact‐angle measurements, four sample states were evaluated: unactivated, unactivated after stretching, activated, and activated after stretching. Compared with the unactivated state, activation resulted in a decrease in the contact angle. Similarly, stretching also caused a slight decrease in the contact angle. These results indicate that both activation and stretching alter the surface structure of RBS‐LMS, thereby causing a slight decrease in the contact angle (Figure 5d).

Another important advantage brought by the introduction of electrospinning technology is its excellent breathability. Commercial gel electrodes used for physiological electrical signal monitoring usually lack breathability and water permeability, which can lead to discomfort during long‐term use (Figure 5e).

Commercial gel electrodes and RBS‐LMS were simultaneously attached to the skin surface. After 12 h, slight whitening of the skin was observed beneath the adhesive region of the gel electrode, which may be associated with its relatively poor breathability. In contrast, almost no visible change was observed in the skin covered by RBS‐LMS (Figure S15). Furthermore, RBS‐LMS, electrospun TPU, and TPU film samples were separately placed on the right arm of a volunteer. Thermal images show that RBS‐LMS exhibits relatively favorable heat‐dissipation capability under both resting and exercise conditions (Figure S16), resulting in a slight reduction in skin‐surface temperature and improved wearing comfort [24].

The electrospun structure of RBS‐LMS endows it with good air and water permeability. Four RBS‐LMS samples with different thicknesses were prepared by controlling the electrospinning duration (Figure 5f). The thinnest RBS‐LMS corresponds to an electrospinning time of 1 h, with a water vapor transmission rate (WVTR) of 9320 g/m^2^·d under the conditions of 25°C and 75% relative humidity. As the electrospinning time increases, the thickness of RBS‐LMS gradually increases. When the electrospinning time is 2, 3, and 4 h, the corresponding WVTR values are 8100, 7080, and 6640 g/m^2^·d, respectively. These results indicate that the thickness of RBS‐LMS has a certain impact on its water vapor transmission rate.

In addition to water vapor transmission rate, RBS‐LMS also possesses liquid permeability, which is crucial for physiological monitoring under sweat‐wetted conditions. A red color developer was slowly dropped onto the RBS‐LMS film; after approximately 10 s, the color developer penetrated the electrospun structure and entered the solution below (Figure 5g). At around 20 s, uniform diffusion channels were established, allowing the color developer to continuously penetrate through the RBS‐LMS.

During physiological monitoring, sweat accumulation or infiltration by other liquids is likely to occur, which poses a test for the underwater working capability of the device. A patterned RBS‐LMS was used, and a blue LED was soldered onto the circuit. After power‐on, both the LED and RBS‐LMS were immersed in deionized water. It can be observed that the LED remained continuously lit underwater (Figure 5h). This indicates that both of the main components of RBS‐LMS, namely TPU and liquid metal, can maintain structural stability in water.

To further verify the long‐term underwater stability, eight RBS‐LMS samples of different specifications were immersed in deionized water, and their resistance was measured at specified intervals (Figure 5i). The results show that within 48 h of immersion, the resistance of RBS‐LMS remained stable, its morphology did not change significantly (Figure S17), and the relative change rate was almost zero.

Compared with water, sweat contains a more complex mixture of components. Meanwhile, the liquid environment encountered on the body surface is inherently variable. Four RBS‐LMS samples were immersed in artificial sweat. During 120 h of immersion, the resistance of RBS‐LMS remained essentially stable (Figure S18). Similarly, after exposure to a constant water flow for 120 h, the resistance changes of all four samples remained below 3% (Figure S19). Overall, RBS‐LMS exhibits good robustness and maintains relatively stable electrical conductivity under stretching, water‐flow exposure, and artificial‐sweat immersion (Figure S20), which is important for the reliability of wearable electronic devices.

Both TPU and liquid metal, the important components of RBS‐LMS, have recyclable properties. For the used RBS‐LMS, acetone solution is first used to dissolve the TPU, the framework part of the material, and then the liquid metal dispersion is separated. Finally, the liquid metal and TPU are recycled and processed separately. The recycled TPU and liquid metal can be reprocessed into new RBS‐LMS (Figure 5j). This environment‐friendly processing method has certain application value for addressing the increasingly serious problem of resource waste.

### 2.6 Physiological Electrical Signal Monitoring

**FIGURE 6:**
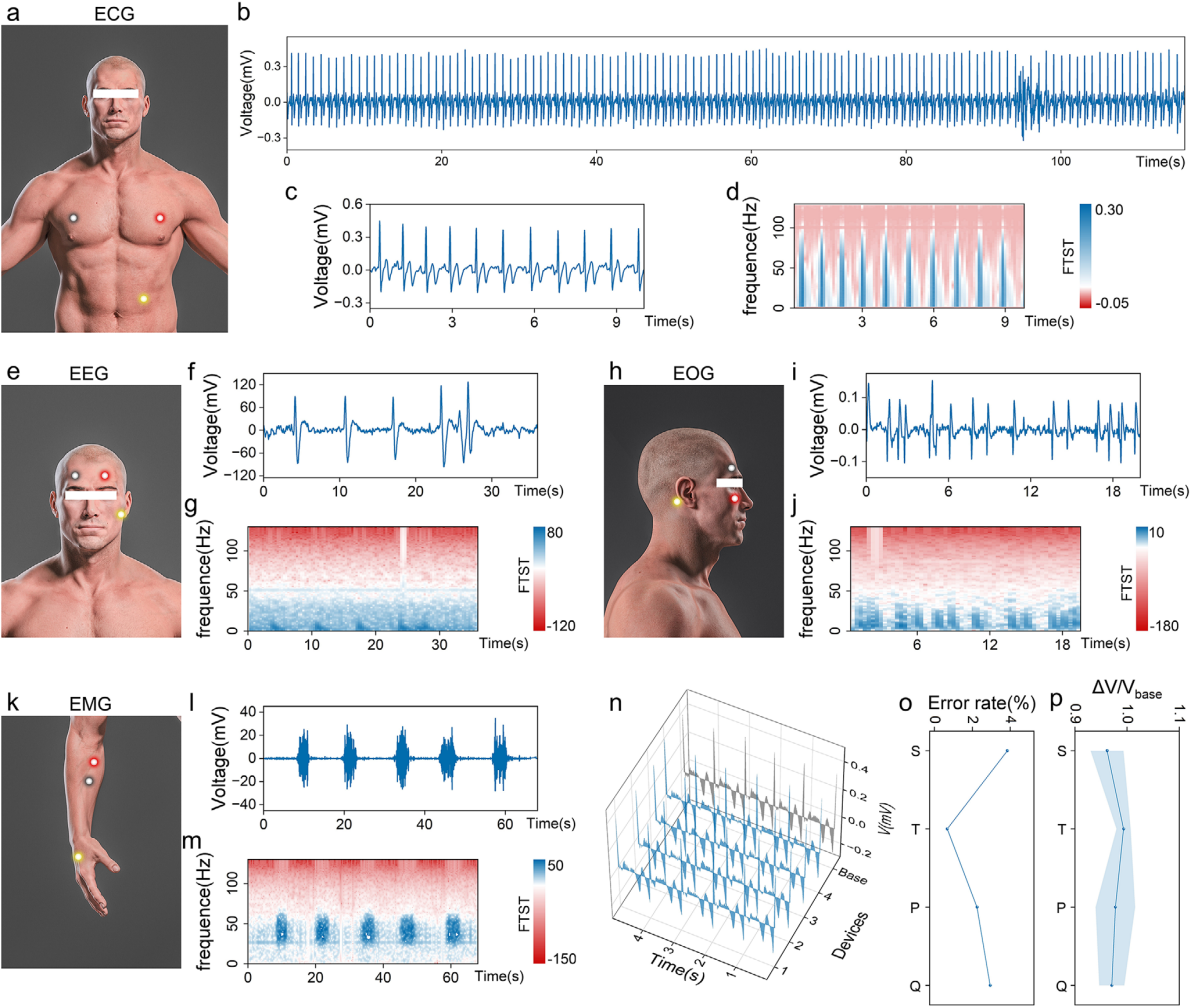
Capability of RBS‐LMS for Physiological Electrical Signal Monitoring. (a) Schematic diagram of RBS‐LMS used for ECG signal monitoring. (b,c) ECG signals of volunteers in a quiet state. (d) Wavelet time‐frequency analysis of ECG signals. (e) Schematic diagram of RBS‐LMS used for EEG signal monitoring. (f,g) EEG signals of volunteers in a quiet state and their wavelet time‐frequency analysis diagrams. (h) Schematic diagram of RBS‐LMS used for EOG signal monitoring. (i,j) EOG signals of volunteers in a quiet state and their wavelet time‐frequency analysis diagrams. (k) Schematic diagram of RBS‐LMS used for EMG signal monitoring. (l,m) EMG signals of volunteers and their wavelet time‐frequency analysis diagrams. (n) Comparison of original signals between RBS‐LMS and commercial electrodes. (o,p) Measurement error of RBS‐LMS. Schematic generated with 3ds Max and Photoshop.

The mechanical toughness, air permeability, and stretchability of RBS‐LMS play an important role in the monitoring of physiological electrical signals. Monitoring electrodes and reference electrodes were arranged on the left and right chest and abdomen respectively (Figure 6a), which can collect the typical stages during heart beating. The typical ECG signals include the QRS complex and T wave (Figure S21). ECG signals were collected when the volunteer was in a working state (Figure 6b). Eleven bands (Figure 6c) were selected for wavelet time‐frequency analysis (Figure 6d), and it can be seen that the periodic high‐energy peak area is obvious, which corresponds to the main distribution interval of the QRS complex. The low‐energy gentle area corresponds to the T wave. Further spectral analysis (Figure S22) and power spectral analysis (Figure S23) of the signals show that the energy of the ECG signals is mainly concentrated in the range of 0–40 Hz, and the signals in other interference frequency bands are relatively weak. This indicates that RBS‐LMS has good anti‐interference ability and can stably collect physiological electrical signals.

To demonstrate the suitability of RBS‐LMS for monitoring physiological bioelectrical signals, we further conducted EEG, EOG, and EMG recordings. For EEG monitoring, the reference and recording electrodes were placed on the cheek and on both sides of the forehead, respectively (Figure 6e). Under this configuration, the EEG signal was dominated by alpha waves. Pronounced signal fluctuations were observed when the volunteer blinked (Figure 6f). The wavelet time–frequency map (Figure 6g) shows that the alpha‐wave frequency is mainly concentrated below 20 Hz; this result is also confirmed in the frequency spectrum (Figure S24) and the power spectral density (Figure S25). By moving the reference electrode behind the ear and positioning the recording electrodes above and below the eye, EOG signals were obtained (Figure 6h). Similar to EEG, blinking exerted a dominant influence on the signal (Figure 6i). The wavelet time–frequency distribution (Figure 6j) shows that the frequency distribution of spontaneous blinks is slightly higher than that of voluntary blinks. Similar results were observed in the frequency spectrum (Figure S26) and the power spectral density (Figure S27).

Unlike passively generated physiological bioelectrical signals, EMG is under voluntary control. To record EMG signals, the reference electrode was placed at the first web space (between the thumb and index finger), and the recording electrodes were positioned on either side of the brachioradialis (Figure 6k). During EMG testing, fist clenching was used to elicit action potentials (Figure 6l). As shown, upon muscle activation, RBS‐LMS recorded the complete signal. The EMG induced by fist clenching exhibited dominant frequency content mainly between 20 and 50 Hz (Figures S28 and S29), and similar results were obtained in the wavelet time–frequency map (Figure 6m).

In the measurement of bioelectrical signals from the skin, impedance is an important parameter. Compared with commercial electrodes, the impedance of RBS‐LMS in its initial state was reduced by approximately 21.92%. After deformation by 80% stretching, folding, and 90° twisting, the impedance of RBS‐LMS remained approximately 28.59%, 24.49%, and 26.86% lower than that of commercial electrodes, respectively (Figure S30). Meanwhile, to evaluate the electromagnetic shielding capability of RBS‐LMS under practical conditions, the sensing performance of commercial electrodes and RBS‐LMS was compared in the presence of AC electrical interference. The signal recorded using the commercial electrodes exhibited obvious interference (Figure S31). In contrast, no obvious interference was observed in the signal recorded using RBS‐LMS. These results demonstrate the ability of RBS‐LMS to shield against electromagnetic interference.

In physiological bioelectrical signal monitoring, stability and reproducibility are also critical. Using commercial gel electrodes as a benchmark, four independent batches of RBS‐LMS were used for ECG measurements (Figure 6n). It can be seen that, compared with commercial gel electrodes, RBS‐LMS captured the key ECG parameters with high fidelity. The relative errors at the four characteristic points (P, Q, S, and T) were all below 4% (Figure 6o,p), demonstrating the stability and accuracy of RBS‐LMS for physiological bioelectrical signal monitoring.

## 3 Conclusions

We report a conductive thin‐film system based on TPU and liquid metal nanofibrous textile. Through simple steps—ultrasonic dispersion, electrospinning, and mold imprinting—we obtained a breathable, stretchable conductive film that maintains stable electrical performance under bending, twisting, and tensile deformation. The film also provides effective shielding against ambient electromagnetic signals. Encapsulation of liquid‐metal microparticles with n‐Butanol effectively mitigates surface‐tension effects and improves biocompatibility. As a multifunctional electronic skin, RBS‐LMS yields stable electrophysiological signals (ECG, EOG, EEG, EMG, etc.) even in complex sweat environments and under electromagnetic interference. These advances are valuable for the further development of high‐performance e‐skins for health monitoring applications.

## Author Contributions

**Cong Huang**: conceptualization, writing – original draft, writing – review and editing, methodology, funding acquisition. **Zhenyu Chen**: methodology. **Zedong Jiang**: data curation. **Yuhao Jiang**: writing – original draft. **Haohong Jiang**: writing – original draft, data curation. **Zhenli Zhou**: writing – original draft. **Yunlu Pan**: writing – original draft. **Shu‐Jen Wang**: writing – original draft, funding acquisition.

## Conflicts of Interest

The authors declare no conflicts of interest.

## Ethical Statement

All volunteers consented and signed an informed consent form prior to the experiments. All experiments were conducted under the review document HIT‐2023061, approved by the Harbin Institute of Technology Ethics Association.

## Supporting information




**Supporting File**: advs78053‐sup‐0001‐SuppMat.docx.

## Data Availability

The data that support the findings of this study are available from the corresponding author upon reasonable request.

## References

[1] A. Chortos , J. Liu , and Z. Bao , “Pursuing Prosthetic Electronic Skin,” Nature Materials 15, no. 9 (2016): 937–950, 10.1038/nmat4671. 27376685

[2] H. Zhang , J. Hong , J. Zhu , et al., “Humanoid Electronic‐Skin Technology for the Era of Artificial Intelligence of Things,” Matter 8, no. 5 (2025): 102136, 10.1016/j.matt.2025.102136.

[3] Q. Zhuang , K. Yao , X. Song , et al., “An ICU‐Grade Breathable Cardiac Electronic Skin for Health, Diagnostics, and Intraoperative and Postoperative Monitoring,” Science Advances 11, no. 12 (2025): adu3146, 10.1126/sciadv.adu3146. PMC11922033 40106548

[4] N. Matsuhisa , D. Inoue , P. Zalar , et al., “Printable Elastic Conductors by in Situ Formation of Silver Nanoparticles from Silver Flakes,” Nature Materials 16, no. 8 (2017): 834–840, 10.1038/nmat4904. 28504674

[5] S. Choi , S. I. Han , D. Jung , et al., “Highly Conductive, Stretchable and Biocompatible Ag–Au Core–Sheath Nanowire Composite for Wearable and Implantable Bioelectronics,” Nature Nanotechnology 13, no. 11 (2018): 1048–1056, 10.1038/s41565-018-0226-8. 30104619

[6] P. Makushko , J. Ge , G. S. Cañón Bermúdez , et al., “Scalable Magnetoreceptive E‐Skin for Energy‐Efficient High‐Resolution Interaction towards Undisturbed Extended Reality,” Nature Communications 16, no. 1 (2025): 1647, 10.1038/s41467-025-56805-x. PMC11828903 39952943

[7] J. Liu , X. Zhang , Y. Liu , et al., “Intrinsically Stretchable Electrode Array Enabled in Vivo Electrophysiological Mapping of Atrial Fibrillation at Cellular Resolution,” Proceedings of the National Academy of Sciences 117, no. 26 (2020): 14769–14778, 10.1073/pnas.2000207117. PMC7334471 32541030

[8] S. K. Kang , R. K. J. Murphy , S. W. Hwang , et al., “Bioresorbable Silicon Electronic Sensors for the Brain,” Nature 530, no. 7588 (2016): 71–76, 10.1038/nature16492. 26779949

[9] I. R. Minev , P. Musienko , A. Hirsch , et al., “Electronic Dura Mater for Long‐Term Multimodal Neural Interfaces,” Science 347, no. 6218 (2015): 159–163, 10.1126/science.1260318. 25574019

[10] C. Linghu , Y. Liu , X. Yang , et al., “Versatile Adhesive Skin Enhances Robotic Interactions with the Environment,” Science Advances 11, no. 3 (2025): adt4765, 10.1126/sciadv.adt4765. PMC11740926 39823320

[11] D. C. Kim , H. J. Shim , W. Lee , J. H. Koo , and D. H. Kim , “Material‐Based Approaches for the Fabrication of Stretchable Electronics,” Advanced Materials 32, no. 15 (2020): 1902743, 10.1002/adma.201902743. 31408223

[12] T. C. Shyu , P. F. Damasceno , P. M. Dodd , et al., “A Kirigami Approach to Engineering Elasticity in Nanocomposites through Patterned Defects,” Nature Materials 14, no. 8 (2015): 785–789, 10.1038/nmat4327. 26099109

[13] K. Sim , Z. Rao , F. Ershad , and C. Yu , “Rubbery Electronics Fully Made of Stretchable Elastomeric Electronic Materials,” Advanced Materials 32, no. 15 (2020): 1902417, 10.1002/adma.201902417. 31206819

[14] T. Someya , Z. Bao , and G. G. Malliaras , “The Rise of Plastic Bioelectronics,” Nature 540, no. 7633 (2016): 379–385, 10.1038/nature21004. 27974769

[15] Y. Ohm , C. Pan , M. J. Ford , X. Huang , J. Liao , and C. Majidi , “An Electrically Conductive Silver–polyacrylamide–alginate Hydrogel Composite for Soft Electronics,” Nature Electronics 4, no. 3 (2021): 185–192, 10.1038/s41928-021-00545-5.

[16] Q. Sun , Y. Zhang , J. Chen , et al., “Highly Stretchable, Moisture‐Permeable, on‐Skin Electrodes from Liquid Metal and Fiber Mat,” InfoMat 7, no. 10 (2025): 70045, 10.1002/inf2.70045.

[17] N. Zhou , J. Ji , R. Qu , et al., “Permeable and Durable Liquid‐Metal Fiber Mat as Implantable Physiological Electrodes with Long‐Term Biocompatibility,” Advanced Materials 37, no. 8 (2025): 2413728, 10.1002/adma.202413728. 39801201

[18] J. Cao , F. Liang , H. Li , et al., “Ultra‐Robust Stretchable Electrode for E‐Skin: In Situ Assembly Using a Nanofiber Scaffold and Liquid Metal to Mimic Water‐to‐Net Interaction,” InfoMat 4, no. 4 (2022): 12302, 10.1002/inf2.12302.

[19] Y. Zhu , R. Haghniaz , M. C. Hartel , et al., “A Breathable, Passive‐Cooling, Non‐Inflammatory, and Biodegradable Aerogel Electronic Skin for Wearable Physical‐Electrophysiological‐Chemical Analysis,” Advanced Materials 35, no. 10 (2023): 2209300, 10.1002/adma.202209300. PMC10006339 36576895

[20] S. Zheng , X. Wang , W. Li , Z. Liu , Q. Li , and F. Yan , “Pressure‐Stamped Stretchable Electronics Using a Nanofibre Membrane Containing Semi‐Embedded Liquid Metal Particles,” Nature Electronics 7, no. 7 (2024): 576–585, 10.1038/s41928-024-01194-0.

[21] T. Lu and F. Chen , “Multiwfn: A Multifunctional Wavefunction Analyzer,” Journal of Computational Chemistry 33, no. 5 (2012): 580–592, 10.1002/jcc.22885. 22162017

[22] X. Hu , Z. Wei , Y. Sun , R. Zhang , and C. Chen , “Multifunctional Bilayer Hydrogel Electronic Skin with Thermochromic and Electromagnetic Interference Shielding for Wearable Sensing Applications,” Composites Communications 53 (2025): 102187, 10.1016/j.coco.2024.102187.

[23] H. R. Lim , H. S. Kim , R. Qazi , Y. T. Kwon , J. W. Jeong , and W. H. Yeo , “Advanced Soft Materials, Sensor Integrations, and Applications of Wearable Flexible Hybrid Electronics in Healthcare, Energy, and Environment,” Advanced Materials 32, no. 15 (2020): 1901924, 10.1002/adma.201901924. 31282063

[24] J. Zhang , S. Yan , Q. An , T. Wu , and L. Zou , “Enhancing Bi‐Directional Thermal Conductivity: A Novel Tri‐Layer Graphene‐Liquid Metal Composite for Advanced Thermal Management,” Composites Communications 55 (2025): 102319, 10.1016/j.coco.2025.102319.

