# Supplementary material for: Robust, Breathable and Stretchable Liquid Metal Composite Nanofibre for Physiological Electrical Signal Monitoring
Source: Adv Sci (Weinh). 2026 Sep 27:e78053. Online ahead of print. doi: 10.1002/advs.78053 (PMC13616267; doi:10.1002/advs.78053)
Supplement: Supplementary file 1 [file ADVS-9999-e78053-s001.docx]

Supporting Information

Robust, Breathable and Stretchable Liquid Metal Composite Nanofibre for Physiological Electrical Signal Monitoring

Cong Huang†*，Zhenyu Chen†，Zedong Jiang†，Yuhao Jiang，Haohong Jiang，Zhenli Zhou，Yunlu Pan*，Shu-Jen Wang*

† These author contributed equally to this work

Dr. C. Huang, Z Chen, Y Jiang, H Jiang, Z Zhou, Prof. S.-J. Wang

Department of Physics, Hong Kong Baptist University, Kowloon Tong, Hong Kong, China

Dr. C. Huang, Z. Jiang, Prof. Y. Pan

State Key Laboratory of Robotics and Systems, School of Mechatronics Engineering, Harbin Institute of Technology, Harbin 150001, China

Dr. C. Huang

Chongqing Research Institute of Harbin Institute of Technology, Chongqing, 401151, China

E-mail:([huangc@hkbu.edu.hk](mailto:huangc@hkbu.edu.hk); [yunlupan@hit.edu.cn](mailto:yunlupan@hit.edu.cn); shujen-wang@hkbu.edu.hk)

**Table of contents**

**Ⅰ Material and method**……………………………………………...………… 1

**Ⅱ Supporting Information**…………………...……………………………… 2

**Figure s1** Comparison of different processing strategies for integrating liquid metals with fiber-spinning techniques.

**Figure s2** Performance comparison with previously reported similar systems.

**Figure s3** Comparison of the particle sizes of liquid metal droplets.

**Figure s4** Dispersion of liquid metal microparticles after different processing treatments.

**Figure s5** Weak interactions between gallium oxide and n-butanol.

**Figure s6** Hirshfeld charge analysis among n-butanol molecules.

**Figure s7** Hydrogen charge analysis between gallium oxide and n-butanol.

**Figure s8** Magnified Raman spectra highlighting the characteristic spectral regions.

**Figure s9** Magnified FTIR spectra highlighting the characteristic absorption regions.

**Figure s10** Resistance changes of RBS-LMS after different levels of tensile deformation.

**Figure s11** Morphological images of RBS-LMS after tensile deformation.

**Figure s12** Effects of thickness and activation state on the electromagnetic shielding performance of RBS-LMS.

**Figure s13** Electromagnetic shielding performance after artificial-sweat immersion, bending, and twisting.

**Figure s14** Flow-channel gap height of RBS-LMS.

**Figure s15** Skin irritation caused by commercial gel electrodes and RBS-LMS.

**Figure s16** Thermal comfort of RBS-LMS, electrospun TPU, and TPU films.

**Figure s17** Underwater structural and electrical stability of RBS-LMS.

**Figure s18** Resistance changes of RBS-LMS after immersion in artificial sweat.

**Figure s19** Resistance changes of RBS-LMS after water-flow rinsing.

**Figure s20** Effects of stretching, water-flow rinsing, and sweat immersion on electrical resistance.

**Figure s21** Schematic illustration of typical ECG signals.

**Figure s22** Frequency spectrum of ECG signals.

**Figure s23** Power spectral distribution of ECG signals.

**Figure s24** Frequency spectrum of EEG signals.

**Figure s25** Power spectral distribution of EEG signals.

**Figure s26** Frequency spectrum of EOG signals.

**Figure s27** Power spectral distribution of EOG signals.

**Figure s28** Frequency spectrum of EMG signals.

**Figure s29** Power spectral distribution of EMG signals.

**Figure s30** Impedance of commercial gel electrodes and impedance changes of RBS-LMS after stretching, folding, and twisting.

**Figure s31** Comparison of the sensing performance of commercial gel electrodes and RBS-LMS under AC electrical interference.

**Ⅲ Reference**…………………...………………………….……………………… 3

Experimental section

Materials

Eutectic gallium–indium (EGaIn, Ga:In=75:25 wt%) was obtained from Dongguan JinXi Co. Polyvinyl pyrrolidone (PVP, 58000), n-butanol (99.5%) and DMF (99%) were purchased from Shanghai Aladdin. TPU was obtained from BASF.

Fabrication and activation of RBS-LMS

Liquid metal and n-butanol were mixed at a mass ratio of 1:10 and sonicated using an AS-650 ultrasonic processor at 90% power with pulsed operation (3 min on, 1 min off) for a total of 16 min. The resulting suspension was mixed at a 1:1 volume ratio with a 15% TPU/10% PVP solution (in DMF) and stirred at 800 rpm for 1 h. The mixture was then electrospun at 12 kV with a 15 cm tip-to-collector distance, using release paper or a silicon wafer as the collector. Film thickness was controlled by the electrospinning duration. An Tuozhu X2D printer was used to fabricate embossed circuit molds with different spacings. The as-spun RBS-LMS was placed on top of the molds, and imprinting was performed by applying prescribed forces scaled to the mold area. After imprinting, conductive pathways formed within the imprinted regions of the RBS-LMS.

Characterization of RBS-LMS

Sample morphology was examined using a Leica DVM6 microscope. Raman spectra were collected using a home-built setup. FTIR spectra were recorded on a Thermo Fisher Nicolet iS50. Electromagnetic shielding performance was evaluated with a PNA-N5244A network analyzer using a two-band setup spanning 1–18 GHz. Electrophysiological signals were acquired using a custom-built system. All experiments involving human participants were approved by the Ethics Committee of Harbin Institute of Technology (HIT-2023061), and written informed consent was obtained from all participants.

First Principles Calculation

All calculations in this work had been performed using the cp2k 7.1 and Multiwfn 3.8. The POTENTIAL density functional is utilized with the BASIS_MOLOPT basis set to calculate structure relax, molecular energy, and electronic charge density differences. The plane-wave cutoff of the grid set is 350 Ry. The structures for electronic charge density differences analysis are the lowest energy states comparing to their other isomers. Gamma scheme was used for the calculations of all the systems. For all complexes, the calculation box is 12.1*12.2*24.3Å.

**Figure s1** Comparison of different processing strategies for integrating liquid metals with fiber-spinning techniques. Schematic generated with 3ds Max and Photoshop.

**Figure s2** Performance comparison with previously reported similar systems.

**Figure s3** Comparison of the particle sizes of liquid metal droplets.

**Figure s4** Dispersion of liquid metal microparticles after different processing treatments.

**Figure s5** Weak interactions between gallium oxide and n-butanol. Schematic generated with Photoshop.

**Figure s6** Hirshfeld charge analysis among n-butanol molecules. Schematic generated with Photoshop.

**Figure s7** Hydrogen charge analysis between gallium oxide and n-butanol.

**Figure s8** Magnified Raman spectra highlighting the characteristic spectral regions.

**Figure s9** Magnified FTIR spectra highlighting the characteristic absorption regions.

**Figure s10** Resistance changes of RBS-LMS after different levels of tensile deformation.

**Figure s11** Morphological images of RBS-LMS after tensile deformation.

**Figure s12** Effects of thickness and activation state on the electromagnetic shielding performance of RBS-LMS.

**Figure s13** Electromagnetic shielding performance after artificial-sweat immersion, bending, and twisting.

**Figure s14** Flow-channel gap height of RBS-LMS.

**Figure s15** Skin irritation caused by commercial gel electrodes and RBS-LMS.

**Figure s16** Thermal comfort of RBS-LMS, electrospun TPU, and TPU films.

**Figure s17** Underwater structural and electrical stability of RBS-LMS.

**Figure s18** Resistance changes of RBS-LMS after immersion in artificial sweat.

**Figure s19** Resistance changes of RBS-LMS after water-flow rinsing.

**Figure s20** Effects of stretching, water-flow rinsing, and sweat immersion on electrical resistance.

**Figure s21** Schematic illustration of typical ECG signals.

**Figure s22** Frequency spectrum of ECG signals.

**Figure s23** Power spectral distribution of ECG signals.

**Figure s24** Frequency spectrum of EEG signals.

**Figure s25** Power spectral distribution of EEG signals.

**Figure s26** Frequency spectrum of EOG signals.

**Figure s27** Power spectral distribution of EOG signals.

**Figure s28** Frequency spectrum of EMG signals.

**Figure s29** Power spectral distribution of EMG signals.

**Figure s30** Impedance of commercial gel electrodes and impedance changes of RBS-LMS after stretching, folding, and twisting.

**Figure s31** Comparison of the sensing performance of commercial gel electrodes and RBS-LMS under AC electrical interference.

**Reference**

[1]	M. Wang, C. Ma, P. C. Uzabakiriho, X. Chen, Z. Chen, Y. Cheng, Z. Wang, G. Zhao, *ACS Nano* **2021**, *15*, 19364.

[2]	J. Li, Y. Zhao, X. Zhao, W. Zhai, K. Dai, C. Liu, C. Shen, *Compos. Part Appl. Sci. Manuf.* **2024**, *179*, 108031.

[3]	K. Chang, M. Luo, *ACS Appl. Eng. Mater.* **2024**, *2*, 2984.

[4]	R. Guo, T. Li, C. Jiang, H. Zong, X. Li, C. Wan, H. Yu, X. Huang, *Adv. Fiber Mater.* **2024**, *6*, 354.

[5]	Q. Zhuang, K. Yao, C. Zhang, X. Song, J. Zhou, Y. Zhang, Q. Huang, Y. Zhou, X. Yu, Z. Zheng, *Nat. Electron.* **2024**, *7*, 598.

[6]	Q. Wang, Y. Sun, C. Qin, Y. Lin, T. Fang, C. Yang, J. Zhang, Y. Lu, D. Kong, *Adv. Mater.* **2025**, *37*, 2417799.

[7]	Q. Sun, Y. Zhang, J. Chen, J. Yang, Y. Xu, P. Zhou, S. Qin, Y. Wang, Z. Wang, J. Wu, H. Haick, Y. Wang, *InfoMat* **2025**, *7*, e70045.

[8]	S. Li, P. Luo, H. Lu, H. Xu, T. Zhou, *Chem. Eng. J.* **2025**, *515*, 163372.

[9]	N. Zhou, J. Ji, R. Qu, X. Feng, X. Song, M. Chen, F. Chen, Z. Ma, Y. Wei, *Adv. Mater.* **2025**, *37*, 2413728.

[10]	S. Zheng, X. Wang, W. Li, Z. Liu, Q. Li, F. Yan, *Nat. Electron.* **2024**, *7*, 576.

[11]	Y. Chen, T. Feng, M. Peng, F. Qin, *Adv. Fiber Mater.* **2025**, *7*, 633.
